# Motivation, barriers, and willingness to participate in clinical trials for novel cancer treatments among the Vietnamese population

**DOI:** 10.1371/journal.pone.0331250

**Published:** 2025-08-29

**Authors:** Pol Van Nguyen, Thanh Thi Thuy Tran, Hieu Thi Thanh Nguyen, Hien Thi Bich Tran, Van Nguyen Thanh Phan, Tram Nguyen Nguyet Luu, Bon Huu Huynh, Khoa Dang Nguyen, Trung Quang Vo

**Affiliations:** 1 Faculty of Pharmacy, Pham Ngoc Thach University of Medicine, Ho Chi Minh City, Vietnam; 2 Social, Economic and Administrative Pharmacy (SEAP) Graduate Program, Faculty of Pharmacy, Mahidol University, Bangkok, Thailand; 3 Faculty of Medicine, Pham Ngoc Thach University of Medicine, Ho Chi Minh City, Vietnam; 4 Faculty of Pharmacy, Hue University of Medicine and Pharmacy, Hue University, Hue, Vietnam; 5 Department of Pharmacology - Clinical Pharmacy, Da Nang University of Medical Technology and Pharmacy, Danang, Vietnam; 6 Interventional Cardiology Department, Gia Dinh People Hospital, Ho Chi Minh City, Vietnam; University of Bergen: Universitetet i Bergen, NORWAY

## Abstract

**Background:**

Novel therapeutic approaches are actively explored and evaluated, but applying these innovations to practice depends critically on the recruitment of volunteers for clinical trials. This study aimed to assess the motivations, barriers, and willingness of the general population in Vietnam to participate in clinical trials for novel cancer treatments.

**Methods:**

This analytical cross-sectional research involved the distribution of self-administered online and printed questionnaires to residents across central and southern Vietnam in December 2024. A structured 23-item questionnaire was developed based on a literature review of studies from the UK, US, and Germany. Items were culturally adapted for Vietnam and translated using a WHO-adapted four-step process, including forward translation, expert panel review, cognitive interviews with 25 participants, and final revision. Face and content validity were assessed during this process. Logistic regression analysis was conducted to determine the factors associated with the respondents’ willingness to participate in the aforementioned clinical trials.

**Results:**

The questionnaires were completed by 851 participants, with the majority aged 40–50 years (30.3%). The respondents also indicated a preference for treatments with prior clinical use and coverage by health insurance (65.6%). The primary motivation for participation was intensive monitoring of conditions (83.7%). The most frequently cited barrier were concerns regarding the high risk associated with less-tested treatments (74.1%). A total of 33.6% expressed a willingness to participate in clinical trials, but fewer (18.9%) were willing to allow such involvement for their children or other family members. Finally, Kinh ethnicity, good health, and positive attitudes toward novel cancer treatments were significantly associated with an increased willingness to participate in clinical trials (p < 0.05).

**Conclusion:**

This study provides critical insights into the motivation of and barriers to participation in clinical trials for novel cancer treatments. Addressing barriers and promoting motivations can contribute to supporting cancer clinical trial conductions and then improving cancer treatment effectiveness in Vietnam.

## Introduction

Cancer remains a major global health burden, as evidenced by a report released by the World Health Organization (WHO), which recorded approximately 20 million new cancer cases and 9.7 million cancer-related deaths in 2022 [1]. That same year, 53.5 million individuals [1] were living within five years of a cancer diagnosis; the most common type of cancer worldwide, with 2.5 million new cases (12.4% of all cancers) recorded, was lung cancer, followed by breast cancer (2.3 million cases, 11.6%), colorectal cancer (1.9 million cases, 9.6%), prostate cancer (1.5 million cases, 7.3%), and stomach cancer (970,000 cases, 4.9%).

In terms of location, Asia recorded the highest cancer burden, with 23.4 million existing cases, 9.8 million new cases, and 5.6 million deaths within five years, as reported by GLOBOCAN in 2022 [2]. To this composition, Southeast Asia contributed substantially, with 2.9 million existing cases, 1.1 million new cases, and 716,000 deaths occurring in the region over a span of five years. A similar situation has plagued Vietnam, with cancer incidence and mortality increasing rapidly in the country. For example, the year 2020 saw Vietnam rank 90^th^ out of 185 countries in terms of cancer incidence – an increase of nine positions from 2018 [3]. It ranked 50^th^ out of 185 countries with respect to cancer-related mortality – up by six positions from 2018 [3], during which an estimated 182,500 new cases and 122,700 deaths from cancer as well as incidence and mortality rates of 159 and 106 per 100,000 people, respectively, were also recorded.

Cancer has emerged as a major challenge, especially in developing countries. More specifically, cancer and cancer treatment reduce productivity, unemployment, labor loss, and capital investment. The estimated global economic cost incurred from 29 types of cancer in 204 countries and territories for the period 2020–2050 is US$ 25.2 trillion (based on 2017 prices), which is equivalent to a reduction of 0.55% in the global gross domestic product [4]. The heaviest economic burden due to cancer is borne by China and the United States, whose shares of costs account for 24.1% and 20.8% of total global levels, respectively.

Current cancer treatments include surgery, radiotherapy, and systemic interventions (chemotherapy, immunotherapy, and targeted therapy). These methods, however, often lack specificity and cause serious side effects, necessitating the development of novel therapeutic strategies [5]. The new approaches explored include hormone therapy, combination therapy, cancer vaccination, the administration of small molecule inhibitors, nanotechnology, cell-based immunotherapy, the use of ferroptosis-targeted drugs, and immunotherapy [6]. Immunotherapy has been an effective treatment for lung and kidney cancers as well as melanoma, as it leverages the body’s immune system so that it attacks cancer cells. Cancer vaccines, such as mRNA-4157 (V940), have been investigated for use in combination with pembrolizumab to treat melanomas and solid tumors, with this intervention showing promising results in reducing recurrence and metastasis [7]. Similarly, nanotechnology enables precise drug delivery, and chimeric antigen receptor T-cell therapy (CAR-T) has achieved notable success as treatment for B-cell leukemia [6]. Potential strategies for enhancing immune response and combatting drug resistance include ferroptosis-targeted medications, such as sorafenib and lapatinib.

These new treatments are evaluated through clinical trials, which are crucial steps before the wide adoption of interventions. This transition from preclinical research to clinical practice follows a structured process that encompasses first-in-human trials (phase I) to post-marketing studies (phase IV). However, the success of clinical trials depends on the recruitment of volunteers, whose participation is driven by various motivations, including contributing to medical advancements, accessing innovative treatments, and helping future patients [8,9]. Such participation can be discouraged by barriers that include long travel times, perceived lack of personal benefit, time constraints, distrust, and limited knowledge about clinical trials [10].

In Vietnam, no study has assessed the (1) willingness of individuals to participate in clinical trials for new cancer treatments. Additionally, understanding the (2) motivators of and (3) barriers to participation is essential for the design of effective recruitment strategies for cancer clinical trials in future. Accordingly, we assessed the three aforementioned variables to illuminate the determinants of participation in clinical trials in the Vietnamese context.

## Methods

### Study design

This analytical cross-sectional study entailed the administration of online and printed self-administered questionnaires to residents of central and southern Vietnam from December 1 to December 31, 2024.

### Sampling and eligibility criteria

Prospective participants were recruited through convenience sampling based on the following inclusion criteria: (1) adults aged 18 years or older who could read and understand Vietnamese, (2) individuals with no cognitive impairments, and (3) people who could communicate normally in Vietnamese. Those who provided incomplete questionnaire responses, withdrew from the survey, or submitted responses that were uniform or followed a repetitive pattern throughout the questionnaire were excluded from analysis.

#### Sample size.

The minimum sample size was calculated using the following formula [11]:


$$\text{n}=\frac{NZ^{\text{2}}P(\text{1}-P)}{d^{\text{2}}(N-\text{1})+Z^{\text{2}}P(\text{1}-P)}=\text{385}$$

where *n* is the minimum sample size, *N* denotes the population of central and southern Vietnam (*N* = 61,132,414: 2024 estimate of the General Statistics Office of Vietnam) [12], P represents the expected proportion of the population who are willing to participate in the research (*P* = 50%), Z refers to the coefficient corresponding to a 95% confidence interval (CI) (*Z* = 1.96), and *d* is the margin of error (*d* = 5%). The result was a minimum required sample size of 385 participants. To account for potential nonresponse and incomplete data, a 10% contingency was applied, which yielded a final required sample size of 424 individuals.

### Data collection

#### Measures.

Data was collected through an online Google Forms^®^ questionnaire distributed through various channels, including email and social networking platforms. To ensure the integrity of the data, the Google Forms^®^ setting to restrict users to a single submission was enabled. Once the participants accessed the questionnaire, they were provided with an informed consent form that they were required to fill out before completing the questionnaire. Individuals who denied consent were directed to the “submit” button but without enabling a perusal of the questionnaire. The same survey was administered offline using a paper-based questionnaire, which trained collaborators distributed to people in high-traffic locations, including major roads, parks, supermarkets, and school entrances. As with the online survey, informed consent was obtained from prospective participants before they were instructed to complete the questionnaire.

#### Questionnaire development.

A literature review of studies conducted in the United Kingdom, the United States, and Germany on cancer patients’ motivations, barriers, and willingness to participate in clinical trials was performed [8–10]. Relevant questions were synthesized and culturally adapted to the Vietnamese context. The resulting structured, anonymous, self-administered questionnaire consisted of 23 items divided into two sections: Section 1 (14 items) addressed motivations, barriers, and willingness to participate in a clinical trial for a new cancer treatment; Section 2 (9 items) covered sociodemographic characteristics. A summary of the questionnaire structure is presented in **Table 1**. Willingness to participate was measured using a 5-point Likert scale ranging from “definitely not” to “definitely participate.” Responses of “definitely not,” “probably not,” and “maybe” were categorized as unwilling, while “probably” and “definitely participate” were categorized as willing to participate.

**Table 1:** Questionnaire sections and measurement scales.

Section		Number of Items	Measurement Scale
(1) Motivations, barriers, and willingness to participate	Motivations	5 items	Single-choice
	Barriers	7 items	Single-choice
	Willingness to participate	2 items	5-point Likert scale
(2) Sociodemographic characteristics		9 items	Single-choice/ Multiple-choice

The questionnaire was translated following a four-step process adapted from WHO guidelines [13]: (1) two independent forward translations, one by a professional translator; (2) expert panel review by five pharmacists from relevant fields; (3) cognitive interviews with 25 pilot participants to assess comprehension and clarity; and (4) revisions based on feedback to finalize the instrument. Face and content validity were established through expert review and pilot testing. As the survey did not include scoring, a reliability assessment was not required.

The developed questionnaire was then translated on the basis of conceptual equivalence using a four-step process adapted from the WHO [13]:

(1) Two independent forward translations, of which the initial translation from the original English version into Vietnamese was performed by a professional translator;
(2) Review by an expert panel composed of five pharmacists practicing in different fields relevant to the study; the panel members provided advice and possible solutions to issues identified in the initial translation;
(3) Cognitive interviews to assess the level of reception of the questionnaire by 25 pilot respondents;
(4) Revision based on feedback and suggestions from the respondents to produce the final version of the questionnaire.

Face validity and content validity were determined in steps 2 and 3. Since the survey did not involve scoring, a reliability assessment was not necessary. The final structured and anonymous self-administered questionnaire consisted of 23 items, among which 14 focused on motivations, barriers, and willingness to participate in a clinical trial on a new cancer treatment and 9 items were devoted to sociodemographic characteristics.

#### Procedures.

To contextualize the questions in the section on motivations, barriers, and willingness to participate, the participants were presented with the following scenario:

Imagine having been diagnosed with cancer and experiencing associated pain. If a clinical trial for a new cancer treatment were to be conducted in Vietnam, would you be willing to participate? What factors would motivate or discourage you from taking part in the trial?

To ensure clarity, we included explanations of the following treatments in the questionnaire: (1) conventional widely used cancer treatments (i.e., surgery, radiation therapy, and chemotherapy) along with (2) newer interventions that integrate traditional methods and emerging therapies (i.e., hormone, combination, and targeted therapies, such as immunotherapy and cancer vaccination; the administration of small molecule inhibitors; the use of nanotechnology in cancer therapy; cell-based immunotherapy; and the administration of drugs targeting the ferroptosis pathway). The participants were explicitly informed that, for the purpose of this study, “new cancer treatment methods” refer to interventions developed more recently than traditional approaches.

The section on sociodemographic characteristics, which was intended to collect basic demographic and health-related information on the participants, was developed on the basis of previous research on the motivations, barriers, and willingness to participate in clinical trials for new cancer treatments [8–10]. The questions in this section covered data such as year of birth, gender, ethnicity, marital status, highest level of education attained, occupation, health insurance status, self-reported health status, and attitudes toward new treatment methods.

### Data processing and statistical analyses

The data from the Google Forms^®^ questionnaires were automatically recorded in Google Sheets, after which the responses were carefully reviewed prior to entry into Microsoft Office 365 to ensure that informed consent was appropriately obtained and that the surveys were thoroughly completed. Fully completed questionnaires were downloaded and exported into Office 365 for data cleaning. A total of 12 questionnaires were eliminated because the respondents provided the same answer to all the questions. To maintain confidentiality, the data were coded before transcription into Microsoft Office 365 for further analysis.

Analyses were conducted using the Statistical Package for the Social Sciences^®^ (Version 26.0, Armonk, NY: IBM Corp., 2019) [14], in which categorical variables were summarized as frequencies and percentages. Descriptive statistical analysis was directed to the demographic characteristics, motivations, barriers, and willingness of the respondents to participate in a clinical trial for a new cancer treatment. Bivariate analyses (S1 Table) were conducted to examine the association between individual factors (e.g., motivations and barriers) and the willingness to participate in a clinical trial, with statistical significance set at *p* < 0.05. To control for potential confounding, variables with a *p*-value < 0.2 in the bivariate analyses were included in the multivariable logistic regression models. Logistic regression analysis was carried out, with the willingness to participate in a clinical trial serving as the dependent variable and sociodemographic characteristics treated as the independent variables.

#### Risk of bias assessment.

A risk of bias assessment was conducted using the Appraisal Tool for Cross-Sectional Studies [15] (S2 Table). There were 20 questions that guided the evaluation of the design, analyses, and reporting of processes and findings in this research, each of which required an answer of “yes,” “no,” or “don’t know.” To minimize bias in the assessment, two authors independently evaluated the study. The Appraisal Tool for Cross-Sectional Studies was used to identify whether an attempt to reduce bias was made in this research rather than providing an overall numerical rating of the risk of bias.

#### Ethical considerations.

The study adhered to the ethical principles outlined in the Declaration of Helsinki and relevant national research ethics guidelines. It was approved by the Scientific Research Ethics Committee at Pham Ngoc Thach University of Medicine (No. 1237/TĐHYKPNT-HĐĐĐ). Participation was entirely voluntary, and no incentives were offered to the respondents. Written informed consent was obtained before enrollment, ensuring that they fully understood the study’s objectives, procedures, potential risks, and their right to withdraw at any time without consequences. Participant confidentiality and data privacy were strictly maintained, with all personal information anonymized and securely stored.

## Results

**Table 2** presents the respondent characteristics and the results on their willingness to participate in a clinical trial of a new cancer treatment. The majority of the participants were 40–50 years old (30.3%), and more than half (57.3%) were female respondents. Most of them (95.3%) identified as Kinh, while the remainder belonged to other ethnic groups. The proportion of married respondents (62.7%) was nearly twice that of single, divorced, or widowed individuals (37.3%). More than half (53.7%) held a university degree or higher. The most common occupations were knowledge-based careers (33.8%), and the vast majority (96.4%) had health insurance. A total of 79.6% rated their health as good or excellent, while 14.9% considered themselves in poor health. Over half of the respondents (65.6%) preferred treatments that had been used for some time and were covered by health insurance. Overall, 18.9% of participants were willing to participate in a cancer clinical trial, while 33.6% were willing to allow their children or family members to participate.

**Table 2:** Respondent characteristics and the willingness to participate in a clinical trial of a new cancer treatment.

Variable	n (%)	Variable	n (%)
**Age**		**Health insurance**	
18–30	224 (26.3)	Yes	820 (96.4)
30–40	160 (18.8)	No	31 (3.6)
40–50	258 (30.3)	**Self-reported health status**	
>50	209 (24.6)	Unknown	47 (5.5)
*Range*	18 - 92	Healthy	677 (79.6)
*Mean (SD)*	41.0 (14.0)	Not healthy	127 (14.9)
**Gender**		**Attitudes towards new treatments**	
Male	363 (42.7)	Willing to try new treatments immediately	126 (14.8)
Female	488 (57.3)	Willing to try only treatments that have been used for a while and are covered by health insurance	558 (65.6)
**Ethnicity**		Only use current treatments	138 (16.2)
Kinh	811 (95.3)	Do not use any treatments	29 (3.4)
Other	40 (4.7)	**Would you be willing to participate in a clinical trial?**	
**Family status**		Definitely not to participate	96 (11.3)
Single/Divorced/Widowed	317 (37.3)	Probably not to participate	133 (15.6)
Married	534 (62.7)	Maybe to participate	336 (39.5)
**Education level**		Probably to participate	252 (29.6)
Below University	394 (46.3)	Definitely to participate	34 (4.0)
University and Postgraduate	457 (53.7)	**Would you be willing to let your children or family members participate in a clinical trial?**	
**Occupation**		Definitely not to participate	154 (18.1)
Manual worker	238 (28.0)	Probably not to participate	205 (24.1)
Knowledge worker	288 (33.8)	Maybe to participate	331 (38.9)
Healthcare worker	63 (7.4)	Probably to participate	148 (17.4)
Housework/Retirement/Unemployed	112 (13.2)	Definitely to participate	13 (1.5)
Students and other	150 (17.6)		

**Fig 1:**
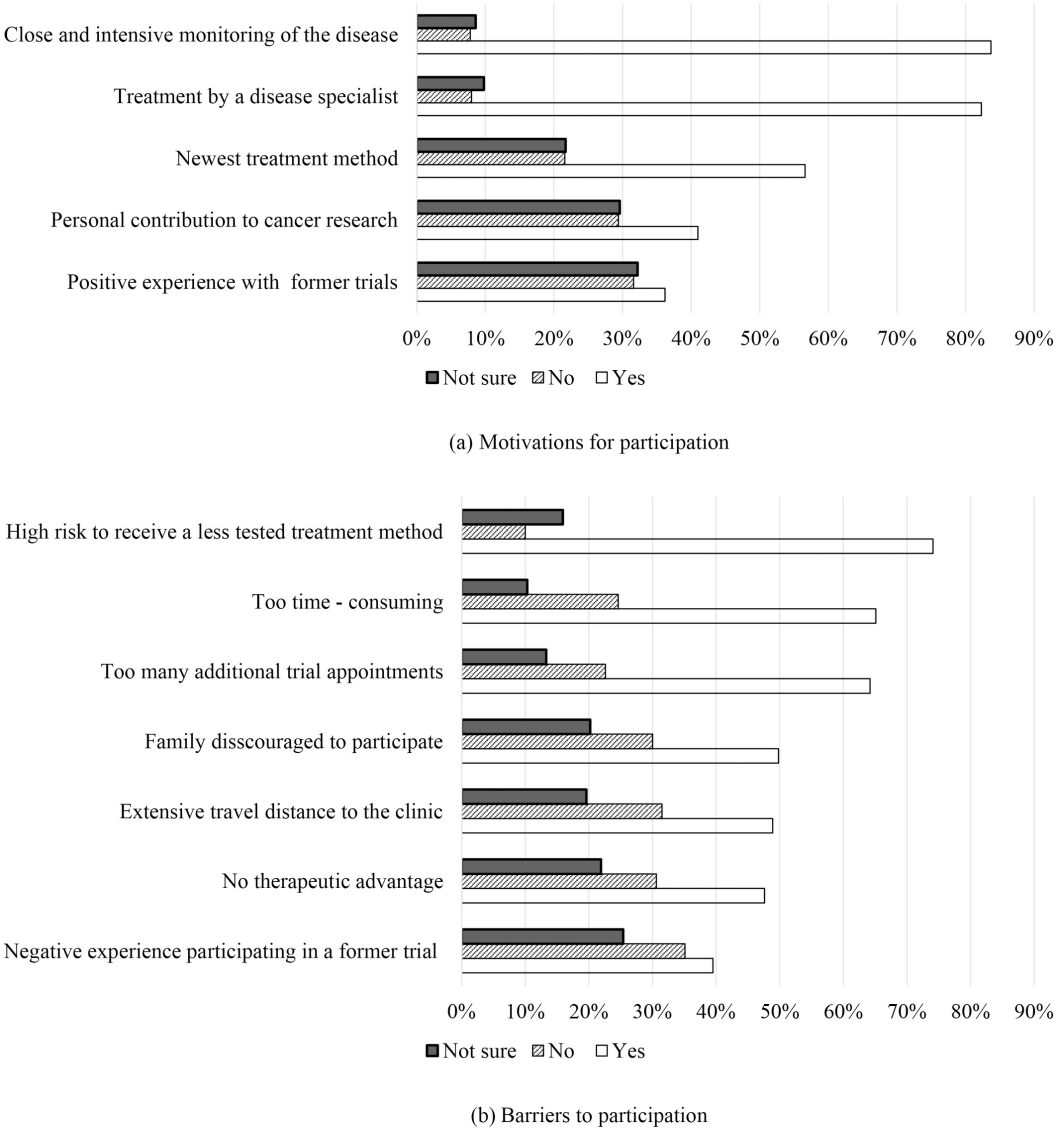
Motivations and barriers to participation in a clinical trial of a new cancer treatment.

**Fig 1** illustrates the respondents’ motivations and barriers to participating in a clinical trial. The most frequently cited motivations were receiving treatment from a disease specialist (82.3%) and benefiting from close and intensive monitoring of their conditions (83.7%). Other motivations included access to the newest treatment methods (56.6%), a personal desire to contribute to cancer research (41.0%), and having a positive experience with previous clinical trials (36.2%). The most common barrier to participation was concerns about the high risk associated with exposure to a less-tested treatment method (74.1%), followed by the perception that trials are excessively time-consuming (65.1%) and the burden of attending too many additional trial appointments (64.2%). Other notable barriers included the lack of therapeutic advantage (47.6%), discouragement by family members (49.8%), extensive travel distance to a clinic (48.9%), and previous negative experiences with clinical trials (39.5%).

Based on bivariate analysis (S1 Table), variables with p < 0.2 were included in multivariable logistic regression to control for confounding. For individual willingness, the model included age, gender, ethnicity, health status, and attitudes toward new treatments. For willingness to allow their children and other family members to participate, the model included age, ethnicity, occupation, and attitudes. **Table 3** shows the adjusted odds ratios (ORs) and 95% confidence intervals (CIs) for factors associated with willingness to participate. Individuals of Kinh ethnicity were significantly more likely to participate in a clinical trial than those from other ethnic groups (OR = 2.7, 95% CI = 1.1–6.5, *p* = 0.026). Self-reported health status was significantly associated with such willingness. Individuals who described themselves as being in good health were nearly twice as likely to take part in clinical trials as those with poor health (OR = 1.9, 95% CI = 1.1–3.0). Individuals who expressed an immediate willingness to adopt a new treatment were significantly more inclined to participate in clinical trials than those who opted for no treatment (OR = 22.8, 95% CI = 5.1–101.3). They were also significantly more likely to let their children or family members participate in a clinical trial, with an OR of 14.2 (95% CI: 3.2–63.1) compared to those who chose not to use any treatment.

**Table 3:** Factors associated with the willingness to participate.

Variables	Themselves		Their children and family members	
	WTP OR (95%CI)*	p-value	WTP OR (95%CI)*	p-value
**Age**				
18 - 30	1.1 (0.7-1.7)	0.755	0.9 (0.4-1.9)	0.696
30 - 40	1.1 (0.7-1.7)	0.799	1 (0.5-1.9)	0.952
40 - 50	1.3 (0.9-2.0)	0.197	1 (0.6-1.7)	0.931
**Gender**				
Male	1.2 (0.8-1.6)	0.348	–	–
**Ethnicity**				
Kinh	2.7 (1.1-6.5)	**0.026**	3.2 (0.9-10.8)	0.065
**Occupation**				
Manual worker	–	–	1.3 (0.6-2.9)	0.572
Knowledge worker	–	–	0.9 (0.4-1.9)	0.742
Healthcare worker	–	–	0.7 (0.3-1.9)	0.465
Housework/Retirement/Unemployed	–	–	1.2 (0.5-3.2)	0.677
**Health status**				
Unknown	0.8 (0.3-1.9)	0.543	–	–
Healthy	1.9 (1.1-3.0)	**0.012**	–	–
**Attitudes towards new treatments**				
Willing to try new treatments immediately	22.8 (5.1-101.3)	**<0.001**	14.2 (3.2-63.1)	**<0.001**
Willing to try only treatments that have been used for a while and are covered by health insurance	5.6 (1.3-24.0)	**0.021**	2.8 (0.6-12.1)	0.174
Only use current treatments	2.4 (0.5-10.8)	0.260	1.7 (0.4-7.8)	0.525

***Note:*** Reference categories: Age > 50, female, other ethnic, students and other, not health, do not use any treatments; *Odds ratios (95% confidence interval; WTP: willingness to participate

**Table 4** shows the association between motivations and the willingness to participate in a clinical trial of a new cancer treatment. Overall, the motivations were significantly associated with the willingness to participate. The respondents who cited treatment by a specialist as motivation were 6.4 times more likely to participate than those who did not mention this motivator (OR = 6.4, 95% CI = 2.7–15). Similarly, those motivated by close and intensive disease monitoring were 5.1 times more inclined to participate than those who were not (OR = 5.1, 95% CI = 2.3–11.4). Significant associations were also found among the respondents willing to have their children or other family members take part in a clinical trial. Motivations such as treatment by a specialist, close monitoring of conditions, and personal contribution to cancer research were significantly associated with willingness, with ORs of 6.1 (95% CI = 1.9–19.7), 5.6 (95% CI = 1.7–17.9), and 5.1 (95% CI = 2.9–8.7), respectively.

**Table 4:** Association between motivations and the willingness to participate.

Motivations	Themselves			Their children and family members		
	n (%)	WTP OR (95% CI)	p-value	n (%)	WTP OR (95% CI)	p-value
**Treatment by a disease specialist**						
No	6 (2.1)	1.0 (ref)		3 (1.9)	1.0 (ref)	
Yes	268 (93.7)	6.4 (2.7-15.0)	**<0.001**	154 (95.7)	6.1 (1.9-19.7)	**0.002**
Not sure	12 (4.2)	1.7 (0.6-4.9)	0.292	4 (2.5)	1.1 (0.2-5.1)	0.906
**Close and intensive monitoring of the disease**						
No	7 (2.4)	1.0 (ref)		3 (1.9)	1.0 (ref)	
Yes	269 (94.1)	5.1 (2.3-11.4)	**<0.001**	149 (92.5)	5.6 (1.7-17.9)	**0.004**
Not sure	10 (3.5)	1.3 (0.5-3.7)	0.579	9 (5.6)	3 (0.8-11.4)	0.117
**Newest treatment method**						
No	30 (10.5)	1.0 (ref)		17 (10.6)	1.0 (ref)	
Yes	218 (76.2)	4.2 (2.8-6.5)	**<0.001**	127 (78.9)	3.5 (2.1-6.0)	**<0.001**
Not sure	38 (13.3)	1.3 (0.8-2.3)	0.295	17 (10.6)	1 (0.5-2.0)	0.987
**Personal contribution to cancer research**						
No	44 (15.4)	1.0 (ref)		17 (10.6)	1.0 (ref)	
Yes	161 (56.3)	4 (2.7-5.9)	**<0.001**	94 (58.4)	5.1 (2.9-8.7)	**<0.001**
Not sure	81 (28.3)	2.2 (1.5-3.4)	**<0.001**	50 (31.1)	3.4 (1.9-6.1)	**<0.001**
**Positive experience with former trials**						
No	54 (18.9)	1.0 (ref)		29 (18)	1.0 (ref)	
Yes	151 (52.8)	3.8 (2.6-5.6)	**<0.001**	97 (60.2)	3.8 (2.4-6.0)	**<0.001**
Not sure	81 (28.3)	1.7 (1.1-2.5)	**0.011**	35 (21.7)	1.2 (0.7-2.0)	0.472

***Note:*** Ref: Reference; *Odds ratios (95% confidence interval); WTP: willingness to participate

The association between the barriers and the willingness to participate in a clinical trial of a new cancer treatment is illustrated in **Table 5**. The barriers to participation were significantly related to the refusal to participate. The respondents who did not perceive time commitment as a barrier were 3.1 times more likely to participate than those who did (OR = 3.1, 95% CI = 2.2–4.4), while those who were uncertain about time commitment were 1.8 times more inclined to take part in clinical trials (OR = 1.8, 95% CI = 1.1–2.9). Similarly, those who did not perceive time commitment as a barrier were 3.4 times more likely to allow their children or other family members to participate (OR = 3.4, 95% CI = 2.3–4.9), while those who were uncertain showed 1.8 times greater likelihood to allow participation (OR = 1.8, 95% CI = 1.0–3.2).

**Table 5:** Association between barriers and the willingness to participate.

Barriers	Themselves			Their children and family members		
	n (%)	WTP OR (95% CI)	p-value	n (%)	WTP OR (95% CI)	p-value
**Too time- consuming**						
Yes	143 (50.0)	1.0 (ref)		72 (44.7)	1.0 (ref)	
No	109 (38.1)	3.1 (2.2-4.4)	**<0.001**	70 (43.5)	3.4 (2.3-4.9)	**<0.001**
Not sure	34 (11.9)	1.8 (1.1-2.9)	**0.013**	19 (11.8)	1.8 (1.0-3.2)	**0.034**
**Too many additional trial appointments**						
Yes	144 (50.3)	1.0 (ref)		78 (48.4)	1.0 (ref)	
No	88 (30.8)	2.4 (1.7-3.3)	**<0.001**	50 (31.1)	2.1 (1.4-3.2)	**<0.001**
Not sure	54 (18.9)	2.6 (1.7-3.9)	**<0.001**	33 (20.5)	2.5 (1.5-4.0)	**<0.001**
**No therapeutic advantage**						
Yes	118 (41.3)	1.0 (ref)		62 (38.5)	1.0 (ref)	
No	115 (40.2)	1.9 (1.4-2.7)	**<0.001**	74 (46)	2.2 (1.5-3.2)	**<0.001**
Not sure	53 (18.5)	1 (0.7-1.4)	0.873	25 (15.5)	0.9 (0.5-1.4)	0.552
**High risk to receive a less tested treatment method**						
Yes	172 (60.1)	1.0 (ref)		93 (57.8)	1.0 (ref)	
No	42 (14.7)	2.6 (1.6-4.1)	**<0.001**	27 (16.8)	2.7 (1.6-4.5)	**<0.001**
Not sure	72 (25.2)	3 (2.1-4.5)	**<0.001**	41 (25.5)	2.5 (1.6-3.9)	**<0.001**
**Family disscouraged to participate**						
Yes	114 (39.9)	1.0 (ref)		49 (30.4)	1.0 (ref)	
No	118 (41.3)	2.3 (1.7-3.2)	**<0.001**	79 (49.1)	3.4 (2.3-5.1)	**<0.001**
Not sure	54 (18.9)	1.2 (0.8-1.8)	0.268	33 (20.5)	1.8 (1.1-2.9)	**0.015**
**Extensive travel distance to the clinic**						
Yes	110 (38.5)	1.0 (ref)		59 (36.6)	1.0 (ref)	
No	112 (39.2)	2 (1.4-2.8)	**<0.001**	56 (34.8)	1.6 (1.1-2.4)	**0.023**
Not sure	64 (22.4)	1.7 (1.2-2.5)	**0.005**	46 (28.6)	2.3 (1.5-3.6)	**<0.001**
**Negative experience participating in a former trial**						
Yes	88 (30.8)	1.0 (ref)		46 (28.6)	1.0 (ref)	
No	110 (38.5)	1.6 (1.2-2.3)	**0.004**	75 (46.6)	2.1 (1.4-3.2)	**<0.001**
Not sure	88 (30.8)	1.9 (1.3-2.8)	**<0.001**	40 (24.8)	1.4 (0.9-2.3)	0.128

***Note:*** Ref: Reference; *Odds ratios (95% confidence interval); WTP: willingness to participate

## Discussion

### Main findings

This study had a total of 851 participants, among whom the majority reported having good health (79.6%) and health insurance (96.4%). When asked about their attitudes toward new treatments, nearly two-thirds were willing to try only treatments that had been used for a while and were covered by health insurance, while a small number preferred to forgo treatment. The willingness to participate in clinical trials of a new cancer treatment (33.6%) was much higher than the willingness to allow the involvement of children or other family members (18.9%). The most frequently mentioned motivations for participation were close and intense monitoring of diseases, treatment by disease specialists, and participation in clinical trials of the newest treatments. The most frequently cited barriers were the risks associated with using less-tested treatments, the time-consuming nature of trials, and excessive additional trial appointments. The findings indicated that ethnicity, health status, and attitudes toward the barriers to and motivations for participation significantly influenced the participants’ willingness to enroll in clinical trials.

### Respondent characteristics

This study targeted the adult general population residing in the central and southern provinces of Vietnam. In contrast, previous studies by Kessel et al. [8] and Sing Yu Moorcraft et al. [9] focused on actual cancer patients. Unlike those studies, our study employed a hypothetical scenario in which participants were asked to imagine themselves as cancer patients experiencing cancer-related pain. This approach enabled them to better understand the questionnaire, provide more practical and contextually relevant responses, and facilitate a more accurate assessment of their preferences, concerns, and decision-making processes in a clinical setting.

Additionally Kessel et al. [8] and Sing Yu Moorcraft et al. [9] relied solely on offline surveys, whereas we employed a mixed-methods approach that incorporated both online and print/paper-based survey formats. The online survey facilitated data collection, storage, and visualization while allowing the participants to complete the questionnaire conveniently using phones or computers in various locations. Nevertheless, online surveys are limited in that they lack direct interactions between respondents and researchers, making it difficult to clarify research objectives or address participant concerns in real time [16]. Online surveys also tend to be more accessible to younger individuals, which can lead to an imbalance in age distribution across respondents [17]. In contrast, offline surveys enable direct interactions, allowing researchers to provide immediate clarification and enhance the accuracy of responses. Accordingly, we combined both online and paper-based survey methods to maximize data collection coverage. This approach helped overcome limitations in certain geographic areas where internet access was limited or unavailable. In these regions, volunteers printed and distributed paper surveys to facilitate participation.

Most of the respondents expressed a preference for therapies that had been in use for some time and were covered by health insurance. In Vietnam, the costs of cancer treatment, particularly out-of-pocket expenses, represent a substantial economic burden. In 2023, expenditures on cancer drugs reached 7,521 billion VND, making them the highest category of total drug payments from the Health Insurance Fund [18]. These findings highlight the critical role of health insurance in alleviating the financial strain stemming from cancer care.

### The willingness to participate in a clinical trial of a new cancer treatment

Of the respondents, 33.6% expressed a willingness to participate in clinical trials themselves, consistent with the findings of Byrne et al., who also found such willingness among 36.5% of their participants [10]. However, a deeper analysis of the current survey results underscored notable differences in willingness between individual- and family-level participation. Specifically, only 18.9% of the respondents wanted their children or other family members to take part in clinical trials, suggesting that while individuals are open to taking risks involving their health, they exhibit greater hesitation when it comes to their loved ones. This phenomenon may be driven by a number of factors. First, personal participation allows individuals to weigh risks and benefits firsthand, whereas entrusting a family member, particularly a child, to a clinical trial introduces additional ethical and emotional considerations. Concerns about potential side effects, the experimental nature of treatments, and uncertainty about long-term outcomes may play a critical role in this reluctance. Second, cultural and familial responsibilities may influence decision-making, as parents and caregivers may prioritize perceived safety and well-being over potential therapeutic benefits when making decisions for their dependents [19–21]. When asked about their willingness to participate in clinical trials, most of the respondents answered “maybe” to both personal participation (39.5%) and participation by their children or other family members (38.9%). The percentages derived here are higher than those reported by Byrne et al. (31.98%) [10], highlighting the importance of addressing public concerns regarding clinical trial participation, particularly for family members. Transparent communication about trial safety, ethical considerations, and the potential benefits and risks may also help mitigate hesitation and foster greater acceptance of participation in clinical research.

### Motivations and barriers to participation

The primary motivation reported by the respondents for participating in clinical trials was close and intensive monitoring of their conditions (83.7%). This suggests that participants placed a high value on the enhanced medical oversight and reassurance they received during the trial process. The appeal of clinical trials for our participants seemed to stem from the immediate personal health benefits, such as early detection of complications and rapid management of side effects, as well as the hope of accessing potentially life-saving or disease-modifying treatments [22]. Kessel et al. [8] found that the most commonly indicated motivation for participating in trials is making a personal contribution to cancer research (74.5%). This indicates a more altruistic motivation, where participants were driven by the wish to help advance scientific knowledge and improve treatment options for future patients, even if they might not directly benefit themselves [23]. This contrast may reflect differences in study populations, cultural contexts, or the way information about the trials was communicated to potential participants.

The preference for treatment by experienced and highly qualified specialists significantly influenced the willingness of the respondents to participate, consistent with the findings of Kessel et al. (21.2%) [8]. Physicians are instrumental in recruiting participants because their ability to clearly and objectively communicate trial information can impact patient decisions. Therefore, ensuring that physicians receive adequate training on how to discuss clinical trials effectively is essential. Providing in-depth consultations without time constraints can also improve patient understanding and willingness to participate. Other key determinants of clinical trial participation are trust in healthcare providers and concerns about conflicts of interest, as highlighted by Chilton et al. [24]. However, in busy hospital environments, where physicians are rotated among different departments and handle heavy workloads, trial recruitment is challenging. Many doctors may be unaware of ongoing clinical trials within their institutions. Addressing this gap requires integrating clinical trial education into medical training from the early stages of a physician’s career. Increased awareness and engagement among all healthcare providers, even those who are not directly involved in research, can facilitate trial recruitment and enhance patient trust. A strong doctor-patient relationship fosters confidence and satisfaction, which in turn may positively influence a patient’s willingness to involve themselves in clinical research [25,26].

The most frequently cited barriers to clinical trial participation were concerns about the risks associated with new treatments (74.1%), the time commitment required for participation (65.1%), and the burden of additional trial appointments (64.2%). Kessel et al. [8] also identified barriers to participation in trials, of which the most prominent were extensive travel time to clinics (25.3%), the absence of therapeutic advantages (24.1%), and excessively time-consuming participation (20.3%). The participants in the present study expressed apprehension over the potential unknown side effects of investigational therapies. Similarly, Meropol et al. reported that patients rank side effects as the most significant impediment to clinical trial participation, with approximately one-quarter believing that they are more likely to experience adverse effects in a clinical trial than with standard treatment [27]. Time constraints (20.3%) and the excessive number of required trial visits (25.3%) were also major deterrents, consistent with the findings of Kessel et al. [8]. Clinical trials often involve frequent follow-up appointments, and travel to and from treatment centers is frequently cited as a reason for nonparticipation [28,29]. This issue is particularly pronounced for individuals living in rural or remote areas, where long travel times and associated costs pose additional burdens [30].

### Factors associated with the willingness to participate

Good health status (OR = 1.9, 95% CI = 1.1–3.0, *p* = 0.012) may be a motivating factor for the willingness to participate in clinical trials, as healthy individuals may perceive themselves as physically capable of handling potential side effects or additional procedures involved in trials. Such individuals might also be more inclined to contribute to medical research, believing that their participation can help advance scientific knowledge and benefit future patients. Conversely, those suffering from poor health may be hesitant due to concerns about the risks of experimental treatments, the potential exacerbation of their conditions, or the burden of additional medical visits. This difference emphasizes the need for tailored recruitment strategies that address both the motivations and concerns of individuals with varying health statuses [31].

The willingness to participate in a new treatment varied among the respondents, particularly when comparing immediate adoption and no history of previous treatment (OR = 22.8, 95% CI = 5.1–101.3). Most patients prioritize finding the most effective treatments for their diseases [28,32,33]. Additionally, individuals who perceive clinical trials as a source of the best possible treatment options are predisposed toward participation [34], implying that attitudes toward new treatments are influenced not only by a general desire for effective care but also by perceptions of clinical research as a pathway to cutting-edge therapies.

Furthermore, the participants who were willing to participate in clinical trials were driven principally by motivations such as receiving treatment from expert physicians (OR = 6.4, 95% CI = 2.7–15). Trust in physicians emerged as the most frequently cited reason for participation, a finding consistent with over a decade of research, reinforcing the criticality of the physician-patient relationship in clinical trial engagement [35]. The motivation to receive close and comprehensive medical monitoring was significantly associated with the willingness to participate, both on an individual level (OR = 5.1, 95% CI = 2.3–11.4) and in decisions involving children and other family members (OR = 5.6, 95% CI = 1.7–17.9). A strong sense of personal contribution to cancer research was also key (*p* < 0.001), suggesting that participation is regarded not only as a means of accessing potential treatment benefits but also as a contribution to scientific and medical advancement. Previous studies conducted on general populations similarly reported a strong belief in the benefits of clinical trials for medical progress and broad support for their implementation [32,36]. These studies discovered that the perception of receiving exceptional medical care in clinical trials significantly influence participation decisions. Conversely, individuals who decline participation tend to cite barriers such as concerns about potential risks, uncertainty regarding treatment effectiveness, fear of side effects, and distrust in experimental therapies. This contrast suggests that individuals with a predominantly positive attitude—characterized by trust in a medical system, belief in the benefits of research, and a desire for outstanding care—are predisposed to involve themselves in clinical trials. Contrastingly, those with negative perceptions of clinical research or heightened concerns about risks are less likely to engage.

Overall, the findings punctuate the need to address both motivational factors and barriers when designing strategies for enhancing clinical trial participation. Strengthening patient trust in physicians, improving awareness of trial benefits, and mitigating concerns about risks through clear, transparent communication are essential to increasing the willingness to participate in new cancer treatment trials. To enhance recruitment and retention, researchers should implement targeted policies that reduce participation barriers while reinforcing principal motivators. Providing easily understandable and accessible information about trial procedures, expected outcomes, and safety measures can help alleviate concerns. Incentives, such as emphasizing the potential for high-quality medical care, access to cutting-edge treatments, and the opportunity to contribute to scientific progress, should be integrated throughout the recruitment process. In addition, leveraging trusted healthcare professionals as messengers can further strengthen patient confidence and engagement. Given that trust in physicians is a dominant motivational factor, direct communication from oncologists and primary care providers may be particularly effective in encouraging enrollment. While access to the best available treatment remains a primary motivator, risks and potential side effects remain major deterrents. Addressing these concerns requires proactive reassurance regarding patient safety, close medical monitoring, and clear risk-benefit communication. Providing educational resources, testimonials from past participants, and opportunities for direct discussion with clinical investigators can likewise build confidence in trial participation [37]. Beyond psychological and informational barriers, logistical challenges must also be considered. Frequent study visits pose a serious burden, particularly for individuals living far from treatment centers. Some trials require weekly or even more frequent visits, which can be a major obstacle for prospective participants [30]. To improve accessibility, researchers should explore flexible scheduling options, offer telemedicine consultations where feasible, and strategically select study sites to minimize travel requirements. These measures can be reinforced with financial support for transportation, lodging, or childcare for individuals grappling with logistical constraints [38]. Furthermore, engaging patient advocacy groups and community organizations can help expand outreach efforts and ensure that diverse populations are adequately represented in clinical research. Tailoring recruitment strategies to address specific demographic and socioeconomic challenges, including language barriers, financial limitations, or cultural beliefs about clinical trials, can foster greater inclusivity. An effective recruitment strategy for cancer clinical trials should combine trust-building measures, clear communication, logistical support, and patient-centered outreach. By comprehensively addressing both motivations and barriers, researchers can enhance participation rates and improve the diversity and generalizability of trial findings.

### Strengths and limitations

This study is the first in Vietnam to examine the motivations, barriers, and willingness of individuals to participate in clinical trials for new cancer treatments. The findings provide valuable insights for policymakers and healthcare professionals, advancing the development of targeted strategies that enhance participation by strengthening motivational factors and addressing potential barriers.

Nevertheless, the limitations of this study should also be acknowledged. We employed a cross-sectional descriptive design, with data collection limited to the central and southern regions of Vietnam. As a result, the findings may not be fully generalizable to the entire Vietnamese population. Additionally, the participants were asked to imagine that they were cancer patients to facilitate their understanding of the questionnaire. Although this scenario helped contextualize the survey, it may not fully capture real-world decision-making processes regarding motivations, barriers, and the willingness to participate in clinical trials.

Future research should expand the study population to include a broader range of participants, particularly diagnosed cancer patients, and extend data collection to all regions of Vietnam. This would improve the generalizability of findings and clear the way for a more comprehensive grasp of motivational and barrier-related factors across different demographic groups. Moreover, because clinical trial participation is influenced by multiple factors beyond those examined in this study, future research should incorporate a wider range of variables to develop more realistic scenarios and better reflect the multifaceted nature of decision-making in clinical trial enrollment.

## Conclusion

This study illuminated the motivations, barriers, and willingness to participate in clinical trials for new cancer treatments in Vietnam. The results have important implications for improving clinical trial recruitment and retention strategies. Such strategies should address both motivations and barriers to enable contribution to a more effective and inclusive clinical trial system, ultimately advancing cancer research and improving patient outcomes in Vietnam.

## Supporting information

S1 Table Bivariate analysis of factors associated with willingness to participate in a clinical trial. (DOCX)

S2 Table Joanna Briggs Institute Critical Appraisal Checklist for Analytical Cross-Sectional Studies. (DOCX)

S3 Table Explanations of checklist items. (DOCX)

S1 File The survey questionnaire. (DOCX)

## Abbreviations

AXIS: Appraisal Tool for Cross-Sectional Studies
CI: Confidence Interval
GDP: Gross Domestic Product
LMIC: Lower-middle income countries
ORs: Odds ratio
WHO: World Health Organization.
