# Supplementary material for: Motivation, barriers, and willingness to participate in clinical trials for novel cancer treatments among the Vietnamese population
Source: PLoS One. 2025 Aug 29;20(8):e0331250. doi: 10.1371/journal.pone.0331250 (PMC12396662; doi:10.1371/journal.pone.0331250)
Supplement: S1 Table — (DOCX) [file pone.0331250.s001.docx]

**S1 Table.** **Bivariate analysis of factors associated with the willingness to participate.**

| **Variable** | **Themselves** | | **Their children and family members** | |
| --- | --- | --- | --- | --- |
|  | **n (%)** | **p-value** | **n (%)** | **p-value** |
| **Age** |  |  |  |  |
| 18 - 30 | 71 (24.8) | **0.199** | 33 (20.5) | **0.195** |
| 30 - 40 | 52 (18.2) |  | 28 (17.4) |  |
| 40 - 50 | 100 (35.0) |  | 56 (34.8) |  |
| >50 | 63 (22.0) |  | 44 (27.3) |  |
| **Gender** |  |  |  |  |
| Male | 132 (46.2) | **0.142** | 68 (42.2) | 0.905 |
| Female | 154 (53.8) |  | 93 (57.8) |  |
| **Ethnicity** |  |  |  |  |
| Kinh | 279 (97.6) | **0.027** | 158 (98.1) | **0.059** |
| Other | 7 (2.4) |  | 3 (1.9) |  |
| **Family status** |  |  |  |  |
| Single/Divorced/Widowed | 104 (36.4) | 0.703 | 56 (34.8) | 0.472 |
| Married | 182 (63.6) |  | 105 (65.2) |  |
| **Education level** |  |  |  |  |
| Below University | 125 (43.7) | 0.281 | 80 (49.7) | 0.338 |
| University and Postgraduate | 161 (56.3) |  | 81 (50.3) |  |
| **Occupation** |  |  |  |  |
| Manual laborer | 36 (16.6) | 0.905 | 54 (33.5) | **0.197** |
| Knowledge worker | 11 (5.1) |  | 47 (29.2) |  |
| Healthcare worker | 41 (18.9) |  | 10 (6.2) |  |
| Housework/Retirement/Unemployee | 81 (37.3) |  | 26 (16.1) |  |
| Students and other | 48 (22.1) |  | 24 (14.9) |  |
| **Health insurance** |  |  |  |  |
| Yes | 277 (96.9) | 0.583 | 156 (96.9) | 0.686 |
| No | 9 (3.1) |  | 5 (3.1) |  |
| **Health status** |  |  |  |  |
| Unknown | 8 (2.8) | **0.001** | 8 (5.0) | 0.576 |
| Excellent | 95 (33.2) |  | 51 (31.7) |  |
| Very good | 34 (11.9) |  | 16 (9.9) |  |
| Good | 120 (42.0) |  | 64 (39.8) |  |
| Not very good | 26 (9.1) |  | 20 (12.4) |  |
| Poor | 3 (1.0) |  | 2 (1.2) |  |
| **Attitudes towards new treatments** |  |  |  |  |
| Willing to try new treatments immediately | 83 (14.7) | **0.000** | 61 (37.9) | **0.000** |
| Willing to try only treatments that have been used for a while and are covered by health insurance | 178 (31.5) |  | 84 (52.2) |  |
| Only use current treatments | 23 (4.1) |  | 14 (8.7) |  |
| Do not use any treatments | 2 (0.4) |  | 2 (1.2) |  |
