# Supplementary material for: Motivation, barriers, and willingness to participate in clinical trials for novel cancer treatments among the Vietnamese population
Source: PLoS One. 2025 Aug 29;20(8):e0331250. doi: 10.1371/journal.pone.0331250 (PMC12396662; doi:10.1371/journal.pone.0331250)
Supplement: S2 Table — (DOCX) [file pone.0331250.s002.docx]

**S2 Table. Joanna Briggs Institute Critical Appraisal Checklist for Analytical Cross-Sectional Studies.**

| **Questions** | | **Yes** | **No** | **Don’t know/comment** | **% Agreement*** |
| --- | --- | --- | --- | --- | --- |
| ***Introduction*** | | | | | |
| 1 | Were the aims/objectives of the study clear? | x |  |  | 100 % |
| ***Methods*** | |  |  |  |  |
| 2 | Was the study design appropriate for the stated aim(s)? | x |  |  | 100 % |
| 3 | Was the sample size justified? | x |  |  | 100 % |
| 4 | Was the target/reference population clearly defined? (Is it clear who the research was about?) | x |  |  | 100 % |
| 5 | Was the sample frame taken from an appropriate population base so that it closely represented the target/reference population under investigation? | x |  |  | 100 % |
| 6 | Was the selection process likely to select subjects/participants that were representative of the target/reference population under investigation? | x |  |  | 100 % |
| 7 | Were measures undertaken to address and categorize non-responders? |  | x |  | 100 % |
| 8 | Were the risk factor and outcome variables measured appropriate to the aims of the study? |  | x |  | 100 % |
| 9 | Were the risk factor and outcome variables measured correctly using instruments/measurements that had been trialed, piloted or published previously? |  | x |  | 100 % |
| 10 | Is it clear what was used to determine statistical significance and/or precision estimates? (e.g., p values, CIs) | x |  |  | 100 % |
| 11 | Were the methods (including statistical methods) sufficiently described to enable them to be repeated? | x |  |  | 100 % |
| ***Results*** | | | | | |
| 12 | Were the basic data adequately described? | x |  |  | 100 % |
| 13 | Does the response rate raise concerns about non-response bias? |  | x |  | 100 % |
| 14 | If appropriate, was information about non-responders described? |  | x |  |  |
| 15 | Were the results internally consistent? | x |  |  | 100 % |
| 16 | Were the results for the analyses described in the methods presented? | x |  |  | 100 % |
| ***Discussion*** | | | | | |
| 17 | Were the authors’ discussions and conclusions justified by the results? | x |  |  | 100 % |
| 18 | Were the limitations of the study discussed? | x |  |  | 100 % |
| ***Other*** | | | | | |
| 19 | Were there any funding sources or conflicts of interest that may affect the authors’ interpretation of the results? |  | x |  | 100 % |
| 20 | Was ethical approval or consent of participants attained? | x |  |  | 100 % |

***Note:*** *Percentage of agreement between two independent reviewers.
