# Supplementary material for: Motivation, barriers, and willingness to participate in clinical trials for novel cancer treatments among the Vietnamese population
Source: PLoS One. 2025 Aug 29;20(8):e0331250. doi: 10.1371/journal.pone.0331250 (PMC12396662; doi:10.1371/journal.pone.0331250)
Supplement: S3 Table — (DOCX) [file pone.0331250.s003.docx]

**S3 Table. Explanations of checklist items.**

Question 1: See **Q1** at the Abstract (last paragraph of Introduction) and end of the Introduction (last paragraph of Introduction).

Question 2: This study used cross-sectional design relating to point-in-time surveys, which is appropriate for the questions being surveyed. See and evaluate **Q2** at Study design of Methods.

Question 3: The minimum sample size was estimated based on the World Health Organization's research sampling formula. See **Q3** at Sample size calculation of Methods.

Question 4: The target population is defined as the the population of Central and Southern Vietnam that satisfies the inclusion and exclusion criteria. See **Q4** at Sample size calculation, Study participants and eligibility criteria, and Data collection of Methods.

Question 5: The sample frame is taken from General Statistics Office of Vietnam in 2024. See **Q5** at Sample size calculation of Methods.

Question 6: See **Q6** at Data collection of Methods. The selection process ensured that the study target population was met, data were collected using self-administered questionnaires through both online and offline methods, using convenience sampling.

Question 7: No measures were taken to address and classify non-respondents because before answering the questionnaire, participants filled out a consent form to participate in the study.

Question 8: The study's questionnaire assessed participants' responses to each question without scoring, so no overall reliability assessment of the questionnaire was conducted.

Question 9: The study's questionnaire assessed participants' responses to each question without scoring, so no overall reliability assessment of the questionnaire was conducted.

Question 10: The statistical methods, software used, and levels of statistical significance (p-values) are clearly stated. See **Q10** at Data processing and statistical analyses of Methods.

Question 11: Methods (including method statistical tables) are fully described. See **Q11** at Data processing and statistical analyses of Methods.

Question 12: Basic data is fully described. See **Q12** at Result.

Question 13: There is no concern about non-response bias, as before answering the questionnaire, participants filled out a consent form and then filled out the entire questionnaire. See **Q13** at Data Collection

Question 14: There is no information about non-respondents because before answering the questionnaire, participants filled out the consent form and then filled out the entire questionnaire.

Question 15: The results are internally consistentl. See **Q15** at Result.

Question 16: The results are presented for all analyses described in the methods. See **Q16** at Result.

Question 17: The author's discussions and conclusions are well supported by the results. See **Q17** at Discussion and Conclusions.

Question 18: Limitations of the study are discussed. See **Q18** at Strengths and limitations.

Question 19: There are no funding sources or conflicts of interest that influence the authors' interpretation of the results.

Question 20: Ethical approval or consent was obtained from participants. See **Q20** at ethical considerations of Methods.
