# Supplementary material for: Motivation, barriers, and willingness to participate in clinical trials for novel cancer treatments among the Vietnamese population
Source: PLoS One. 2025 Aug 29;20(8):e0331250. doi: 10.1371/journal.pone.0331250 (PMC12396662; doi:10.1371/journal.pone.0331250)
Supplement: S1 File — (DOCX) [file pone.0331250.s004.docx]

**S1 File. The survey questionnaire**

| 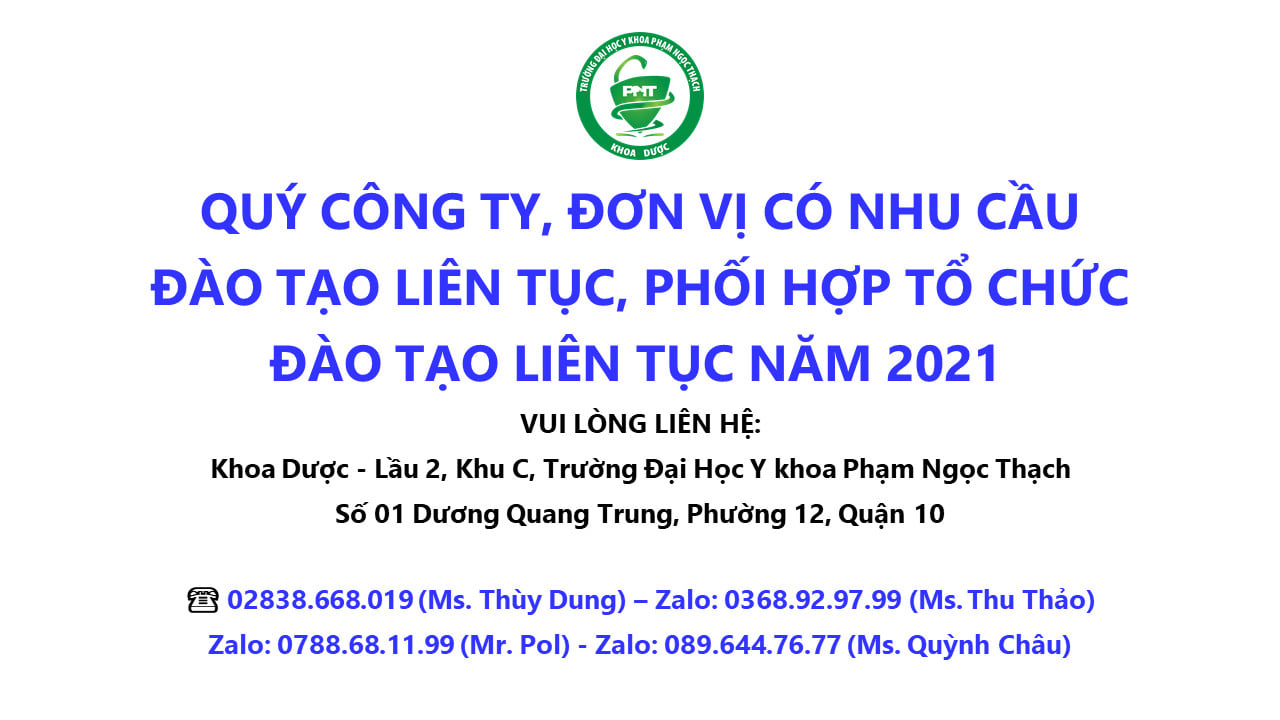 | Code: \|__\|__\|_A_\|__\|__\|__\|  SCIENTIFIC RESEARCH PROJECT  2024-2025 |
| --- | --- |

**INTERVIEWWEE’S CONSENT TO PARTICIPATE FORM**

*Dear Sir/Madam,*

*We respectfully invite you to participate in the study:*

**“Motivation, barriers, and willingness to participate in clinical trials for a new cancer treatment and willingness to pay for mRNA-based cancer vaccines among people in selected provinces and cities in Vietnam.”**

This study aims to examine your motivations, barriers, acceptance, and threshold for payment for mRNA-based cancer vaccines. By participating in this study, all information you provide to us will be kept confidential and will be used for scientific research purposes only. Your participation is completely voluntary, meaning you can agree or disagree to participate, or you can stop participating at any time during the process.

For any questions regarding this study, please contact:

**TRAN THI THUY THANH**

Phone number: **xxxx.xxx.xxx** Email: [xxx@gmail.com](mailto:thanhthanh260320022002@gmail.com)

If you agree to participate in this study, please fill out the following information:

I have read and been clearly explained the purpose, method and content of the research.

**I agree to participate in the study.**

**Full name: …………………………………………Signature:**___________________

**Researcher (or person who explains and forms consent)**

I confirm that I have explained to the volunteer the nature and purpose of the study. I have answered the participant's questions. I have answered the volunteer's questions on the date stated on the consent form.

**Full name: ...........................................................................**

**Signature: _____________________________________**

**Day: |__||__|/|__||__|/2024**

**PART 1. MOTIVATION, BARRIERS AND WILLINGNESS TO PARTICIPATE IN A CLINICAL TRIAL OF A NEW CANCER TREATMENT**

Suppose that there is **a clinical trial of a new cancer treatment** that will be conducted in Vietnam, in your opinion:

1. Do the following **MOTIVATIONS** motivate you to participate in a clinical trial of a new cancer treatment?

| **No.** | **Motivational factors** | **Yes** | **No** | **Not sure** |
| --- | --- | --- | --- | --- |
| 1.1 | Treatment by a disease specialist | ⭘^1^ | ⭘^2^ | ⭘^3^ |
| 1.2 | Close and intensive monitoring of the disease | ⭘^1^ | ⭘^2^ | ⭘^3^ |
| 1.3 | Newest treatment method | ⭘^1^ | ⭘^2^ | ⭘^3^ |
| 1.4 | Personal contribution to cancer research | ⭘^1^ | ⭘^2^ | ⭘^3^ |
| 1.5 | Positive experience with former trials | ⭘^1^ | ⭘^2^ | ⭘^3^ |

2. Do the following **BARRIERS** prevent you from participating in a clinical trial of a new cancer treatment?

| **No.** | **Barrier factors** | **Yes** | **No** | **Not sure** |
| --- | --- | --- | --- | --- |
| 2.1 | Too time - consuming | ⭘^1^ | ⭘^2^ | ⭘^3^ |
| 2.2 | Too many additional trial appointments | ⭘^1^ | ⭘^2^ | ⭘^3^ |
| 2.3 | No therapeutic advantage | ⭘^1^ | ⭘^2^ | ⭘^3^ |
| 2.4 | High risk to receive a less tested treatment method | ⭘^1^ | ⭘^2^ | ⭘^3^ |
| 2.5 | Family disscouraged to participate | ⭘^1^ | ⭘^2^ | ⭘^3^ |
| 2.6 | Extensive travel distance to the clinic | ⭘^1^ | ⭘^2^ | ⭘^3^ |
| 2.7 | Negative experience participating in a former trial | ⭘^1^ | ⭘^2^ | ⭘^3^ |

Please indicate your level of **participation or non-participation** with the following questions on a Likert scale **from 1 to 5**:

1 = Definitely not participating 2 = Probably not participating

3 = Not sure whether to participate or not 4 = Probably participating

5 = Definitely participating

| **3. If a clinical trial of a new cancer treatment required healthy volunteers, would you:** | | **Level of participation** | | | | |
| --- | --- | --- | --- | --- | --- | --- |
|  |  | **1** | **2** | **3** | **4** | **5** |
| 3.1 | … be willing to participate in a clinical trial? | ⭘^1^ | ⭘^2^ | ⭘^3^ | ⭘^4^ | ⭘^5^ |
| 3.2 | …would you be willing to let your children or family members participate in a clinical trial? | ⭘^1^ | ⭘^2^ | ⭘^3^ | ⭘^4^ | ⭘^5^ |

**PART 2. SOCIODEMOGRAPHIC CHARACTERISTICS**

| **No.** | **Questions** | **FEEDBACK OPTIONS** |  |
| --- | --- | --- | --- |
| **4.** | **Year** | \|___\|___\|___\|___\| |  |
| **5.** | **Gender** | O 1.Male | O 2. Female |
| **6.** | **Ethnicity** | Ο 1. Kinh  Ο 2. Hoa  Ο 3. Cham | Ο 4. Kho-me  Ο 5. Others: ……………… |
| **7.** | **Family status** | O 1. Single/Divorced/Widowed | O 2. Married |
| **8.** | **Education level** | Ο 1. Below elementary school  Ο 2. Elementary school  Ο 3. Junior high school  Ο 4. High school | Ο 5. Primary/Intermediate vocational training  Ο 6. College  Ο 7. University  Ο 8. Postgraduate |
| **9.** | **Occupation** | Ο 1. Manual laborer  Ο 2. Knowledge worker  Ο 3. Healthcare worker  Ο 4. Housework | Ο 5. Retirement  Ο 6. Unemployed  Ο 7. Students  Ο 8. Others: ………………. |
| **10.** | **Health insurance** | Ο 1. Yes | Ο 2. No |
| **11.** | **Health status** | Ο 1. Unknown  Ο 2. Excellent  Ο 3. Very good | Ο 4. Good  Ο 5. Not very good  Ο 6. Poor |
| **12.** | **Attitudes towards new treatments** | Ο 1. Willing to try new treatments immediately  Ο 2. Willing to try only treatments that have been used for a while and are covered by health insurance  Ο 3. Only use current treatments  Ο 4. Do not use any treatments | |

**--- Thank you very much for your participation---**
